# Cytogenetic and molecular insight into the genetic background of disorders of sex development in seventeen cats

**DOI:** 10.1038/s41598-022-21718-y

**Published:** 2022-10-24

**Authors:** Monika Stachowiak, Izabela Szczerbal, Joanna Nowacka-Woszuk, Tomasz Nowak, Natalia Sowinska, Anna Lukomska, Maciej Gogulski, Malgorzata Badura, Karolina Sklorz-Mencel, Dariusz Jagodka, Wojciech Nizanski, Stanislaw Dzimira, Marek Switonski

**Affiliations:** 1 Department of Genetics and Animal Breeding, Poznan University of Life Sciences, Wolynska 33, 60-637 Poznan, Poland; 2 Department of Preclinical Sciences and Infectious Diseases, Poznan University of Life Sciences, Poznan, Poland; 3 Centre of Biosciences, Institute of Animal Physiology, Kosice, Slovakia; 4 University Centre for Veterinary Medicine, Poznan University of Life Sciences, Poznan, Poland; 5 Mencel-Vet Veterinary Clinic, Borek Wielkopolski, Poland; 6 Aura Veterinary Clinic, Łochowo, Poland; 7 Department of Reproduction and Clinic of Farm Animals, Wroclaw University of Environmental and Life Sciences, Wrocław, Poland; 8 Department of Pathology, Wroclaw University of Environmental and Life Sciences, Wrocław, Poland

**Keywords:** Developmental biology, Genetics

## Abstract

The genetic background of feline disorders of sex development (DSDs) is poorly understood. We performed comprehensive cytogenetic, molecular, and histological studies of 17 cats with abnormal external genitalia, unusual behavior, or tricolor coats (atypical in males). The DSD phenotype of three cats was associated with sex chromosome abnormalities: X/Y translocation (38,XX^*SRY*+^), 37,X/38,XY mosaicism, and XX/XY leukocyte chimerism. The remaining 14 affected cats were classified as XY DSD (*SRY*-positive). In this group and 38 normal males, we analyzed a priori selected candidate genes (*SRY, TAC3, CYP11B1* and *LHCGR*). Only a previously reported nonpathogenic variant was found in *SRY*. Moreover, *SRY* gene copy number was determined, and three variants were observed: 6, 5 (modal), and 4 copies in a single DSD case. The known variants in *TAC3* and *CYP11B1*, responsible for testicular hypoplasia, persistent primary dentition or congenital adrenal hyperplasia, were not found in the study group. Nine novel polymorphisms were identified in the *LHCGR* gene, one of which, a potentially regulatory indel variant in 5′UTR, was significantly associated (*p* = 0.0467) with XY DSD. Our report confirmed that abnormalities of sex chromosomes are important causes of feline DSDs. We also showed that the indel variant of *LHCGR* can be considered a promising marker associated with XY DSD phenotype.

## Introduction

Disorders of sex development (DSDs) are a serious health issue in mammalian domestic animals, including cats^1,2^. It is well known that DSDs have a negative impact on fertility, increase the risk of gonadal cancer, and can affect behavior. The genetic background of these disorders is heterogeneous and three major categories can be distinguished, based on the observed sex chromosome complement: (a) DSD due to abnormal sex chromosome complement, (b) XX DSD: animals with a normal XX set of chromosomes, and (c) XY DSD: animals with a normal XY set of chromosomes^3^. Primary classification to the appropriate type of DSD is based on the clinical examination of external and internal genitalia, followed by cytogenetic analysis and molecular detection of Y-linked *SRY* gene, which plays a crucial role in male development^1^.

There have been number of reports dealing with sex chromosome abnormalities in DSD cats, including X monosomy, XXY trisomy, XX/XY leukocyte chimerism, 37,X/38,XY mosaicism, and two cases of structural rearrangements involving sex chromosomes—namely 37,X/38,X,r(Y) and 38,XX.ish der(X)t(X;Y)^4–9^. However, the molecular background of DSD in cats with a normal set of XX or XY chromosomes has been poorly elucidated. Although a number of candidate genes were evaluated in XY cats with altered external genitalia (*SRY, AR, SRD5A2, MAMLD1, HSD3B2, HSD17B3, DHH, RSPO1*)^7,10–13^, to date only two causative mutations have been reported. A missense G > A SNP (Arg > Gln) in exon 7 of the *CYP11B1* gene*,* involved in the biosynthesis of adrenal corticoids, has been found to contribute to congenital adrenal hyperplasia and associated gynecomastia, empty scrotum, and aggressive behavior in a tomcat with a normal 38,XY chromosome set^14^. The second causative variant was located in the *TAC3* gene encoding tachykinin 3, which is a precursor of the neurotransmitter neurokinin B. In a domestic shorthair tomcat with testicular hypoplasia, one testicle in the scrotum, primary dentition, and small body size, a homozygous genotype for a missense substitution (c.220G > A, p.Val74Met) was identified^13^. Genetic screening for this variant in a cohort of 171 randomly selected control cats revealed two carriers. It is known that mutations of this gene are also responsible for DSD phenotype in humans^15^.

The Y-linked *SRY* gene is a key regulator of male sex determination in mammals and its mutations are well known causes of DSD in males^16^. So far, only one polymorphism in the coding sequence of this gene has been identified in cats; this is a missense c.389G > C substitution (p.Arg130Thr), but was excluded as a cause of feline DSD based on its distribution^10,17^. On the other hand, there is no information about the variability of *SRY* copy number in cats, while recent study showed that the presence of two copies of the *SRY* gene, rather than the three copies commonly observed in dogs and wolves, may be associated with XY DSD in Yorkshire terriers^18^.

The *LHCGR* gene, which encodes luteinizing hormone/chorionic gonadotropin receptor, is another candidate gene for DSD in mammals. In XY DSD human patients, mutations that inactivate *LHCGR* are responsible for female external phenotype (Leydig cell hypoplasia, type 1) or the incomplete virilization of external genitalia (Leydig cell hypoplasia, type 2), as well as for a range of penile defects such as micropenis and hypospadias^19^. DNA variants of *LHCGR* have not been studied previously in DSD cats.

In this study, we aimed to carry out comprehensive cytogenetic and molecular studies of seventeen DSD cats with abnormal external genitalia, unusual tricolor coats in males, and abnormal sexual behavior. All cats were cytogenetically examined in order to establish their sex chromosome complement, while the molecular analysis focused on four a priori selected candidate genes (*SRY*, *TAC3, CYP11B1* and *LHCGR*).

## Materials and methods

### Animals

Altogether seventeen cats with ambiguous external genitalia, abnormal behavior or tricolor coat selected by veterinarians were included in this study (Table 1). Five DSD cases were previously characterized in our earlier work (Table 1). A total of 38 normal males (Table S1) were taken as a control group. Two females were additionally included as references for a study of X chromosome inactivation, and as controls for molecular analyses of *SRY*. Blood samples and skin fragments from cases #7355 and #7513 were collected in order to establish *in vitro* fibroblast culture during standard veterinary procedures, and with the consent of the owners. Hair follicles were also sampled in case #7513. Most of the DSD cats had been neutered on medical recommendation, and the testes were subjected to histological examination. All procedures were consistent with standard Polish veterinary practices and gained the approval of the local Bioethical Commission for Animal Care and Use in Poznan, Poland (Certificate No. 1/2019). All experiments were carried out in compliance with the ARRIVE guidelines and all methods were performed in accordance with the relevant guidelines and regulations.

**Table 1:** Characteristics of the DSD cats.

Cases (ID)	Breed	Phenotype/histology of gonads	Chromosomes
#7318	Domestic shorthair	Male posture; normal penis; bifid scrotum; tortoiseshell coat pattern/Testes with seminiferous tubules; normal Sertoli cells; numerous Leydig cells; lack of germinal cells	XX^*SRY*+^
#7355	Bengal	Normal male external genitalia; female behavior; one mating resulted one offspring (male): parentage confirmed by analysis of STR markers/Testes with degenerated spermatogenesis; rare sperm	X/XY
#7513	Domestic shorthair	Regular estrus; rudimentary penis; vulva; abdominally located testicles (USG)/Testes; sparse seminiferous tubules; hypertrophic Sertoli cells; numerous Leydig cells; lack of germinal cells	XX/XY
#6533^11^	Persian	Penis restricted to the glans; absence of prepuce; hypospadias; bifid scrotum; imperforate anus and rectourethral fistula/Testes without mature sperm; underdevelopment of Leydig cells	XY
#6664^10^	Domestic shorthair	Rudimentary penis; hypospadias (urethral opening located on dorsal surface of the penis); persistent frenulum; lack of scrotal septum; everted mucosa of rectum surrounding anus; bifid scrotum/Testes with normal spermatogenesis	XY
#6680^10^	Domestic shorthair	Penis with spines; persistent frenulum; blind-ended vulva; everted mucosa of rectum surrounding anus/Testes with normal spermatogenesis	XY
#6699^10^	Domestic shorthair	Rudimentary penis; hypospadias (urethral opening located dorsally to the penis); glans penis not covered by prepuce; lack of scrotal septum; everted mucosa of rectum surrounding anus; testes located under the skin in inguinal position/Testes; lack of sperm	XY
#6856^7^	Domestic shorthair	Vulva and enlarged clitoris with some spines; bifid scrotum; small gonads; lack of uterus (laparotomy inspection)/Testes with numerous seminiferous tubules; lack of spermatogonia; decreased number of Leydig cells	XY
#6721	Crossbred	Underdeveloped penis with rudimentary spines; strongly caudally directed penis; hypospadias (urethral opening located under horizontal mucosal fold between penis and anus); bifid scrotum; reduced semen parameters/Testes; numerous seminiferous tubules; rare sperm	XY
#6973	Domestic shorthair	Rudimentary penis; underdeveloped prepuce/Testes; rare sperm; numerous Leydig cells	XY
#7036	British shorthair	Penile defects: strongly caudally directed penis; glans split; abnormal scrotum resembling a vulva/Testes with normal spermatogenesis	XY
#7145	British shorthair	Male and female behavior; underdeveloped penis and prepuce; phimosis/Testes with numerous seminiferous tubules; rare sperm	XY
#7254	Domestic shorthair	Penis with spines in a position extremely close to anus; hypospadias (urethral opening located in a cavity that resembles the vaginal vestibule); asymmetric gonads located under skin on both sides of penis; frequent urination and defecation/Testes with numerous seminiferous tubules; frequent sperm in the larger and rare sperm in the smaller testicle; numerous Leydig cells	XY
#7302	Domestic shorthair	Penis with spines strongly directed posteriorly, wider than usual; hypospadias (urethral opening covered by deformed penis); mucosa located between penis and the lower part of partially underdeveloped anus; bifid scrotum with symmetric gonads located on both sides of penis; abnormal location of internal organs in abdominal cavity (USG)/Testes with numerous seminiferous tubules and Sertoli cells; frequent sperm; decreased number of Leydig cells	XY
#7320	British	Lack of penis; ambiguous genitalia resembling abnormal vulva/cloaca; anal stricture/NA	XY
#7451	Domestic shorthair	Scrotal hypospadias; bifid scrotum with asymmetric gonads located on both sides of penis/NA	XY
#7455	Ragdoll	Ambiguous genitalia; bent penis with spines; curved penile glans; abnormal prepuce; hypospadias; mucosa surrounding in perineal area and anus; bifid scrotum with asymmetric gonads/Testes with numerous seminiferous tubules, in larger gonad with sperm, in smaller gonad without sperm	XY

*NA* not analyzed.

### Histological analysis

Gonadal samples were fixed in 10% formalin, processed, and embedded into paraffin blocks. The 3 µm serial sections were stained with hematoxylin and eosin. The slides were analyzed on an Axio Lab.A1 microscope (Carl Zeiss Microscopy, Jena, Germany) equipped with an Axiocam ERc5s digital camera (Carl Zeiss Microscopy, Jena, Germany) or on Olympus BX53 light microscope coupled with an Olympus UC90 camera.

### Cytogenetic analysis

Chromosome preparations were obtained from lymphocyte culture or skin fibroblast culture using standard procedures. The chromosome complement was analyzed by Giemsa staining and fluorescence *in situ* hybridization (FISH) with feline X and Y chromosome painting probes (kindly provided by Professor M.A. Ferguson-Smith, Cambridge University, UK) and a locus-specific BAC (RP86-278G21) probe specific to the *SRY* gene, obtained from the Feline BAC library RPCI-86 (https://bacpac.chori.org/). The probes were labelled with biotin-16-dUTP or digoxigenin-11-dUTP by DOP-PCR and detected using streptavidin‐Cy3 (GE Healthcare) (red signal) or anti-digoxigenin-fluorescein (Roche Applied Science) (green signal). The chromosomes were counterstained with Vectashield containing DAPI (Vector Laboratories). Microscopic evaluation was carried out under a Nikon E600 Eclipse microscope (Melville, NY, USA), equipped with a cooled CCD digital camera and Lucia software. For each individual, at least thirty metaphase spreads were analyzed.

### Molecular detection of sex-chromosome-linked genes

Genomic DNA was isolated from peripheral blood using a Blood Mini Kit (A&A Biotechnology) and additionally from fibroblasts for case #7513, with the use of a Genomic Mini Kit (A&A Biotechnology). The Y-linked *SRY* and *ZFY* genes and the X-linked *ZFX* gene were amplified using the primers shown in Table S2^4,20^. The *SRY* gene underwent PCR detection (1022 bp band) and its entire coding sequence was sequenced for the DSD and control cats. The amplicons for *ZFY* and *ZFX* (448 bp) were digested with restriction enzyme (*Bsm*I) for all DSD cats. The use of 2% agarose gel electrophoresis allowed us to distinguish *ZFY* (448 bp band) from *ZFX* (391 bp and 57 bp bands). Moreover, to verify the size of the translocated region from the Y to the X chromosome, we additionally amplified the Y-linked genes *TETY1, TETY2, CUL4BY, CYORF15, HSFY, FLJ36031Y* using the primers shown in Table S2^21^. The selected genes are located on short or long arm of the Y chromosome, as it was shown by Wilkerson et al.^22^ and Li et al.^23^.

### Analysis of X-chromosome inactivation status

DNA from blood was isolated with the use of a MasterPure DNA Purification Kit for Blood (Epicentre). The 2 µg of DNA was digested with methylation-sensitive *Hpa*II restriction enzyme (20U/reaction) at 37 °C for 15 h, followed by heat inactivation at 95 °C for 10 min. The use of *Hpa*II enzyme assured that the active (nonmethylated) X chromosome will be digested, and thus that PCR amplification will be possible only from the inactive (methylated) X chromosome, as described by Mochizuki et al.^24^ The PCR was performed for the fragment (exon 1) of the X-linked androgen receptor (*AR*) gene, in which a short tandem repeat (CAG)_n_ polymorphism is present and the study animals were heterozygous in terms of the number of CAG repeats. Standard amplification conditions (32 cycles, 60 °C for annealing) were used with the primers shown in Table S2. The amplicons were diluted with water (1:4 ratio) and 1 µl was mixed with formamide (8.8 µl) and GeneScan 500 LIZ Dye Size Standard (0.2 µl) for capillary electrophoresis on a Genetic Analyzer 3130 (Applied Biosystems). The area under the peak (representing amplified alleles of the *AR* gene) was measured using ImageJ software and calculated for particular peaks as a percentage from a sum for both alleles, which represented 100%. The analysis was performed for the 38,XX^*SRY*+^ case (#7318) and for two control healthy females.

### Parentage verification and chimerism analysis

Genotypes for fourteen STR (short tandem repeats) markers (FCA149, FCA441, FCA229, FCA678, FCA075, FCA026, FCA220, FCA453, FCA310, FCA201, FCA105, FCA649, FCA069, FCA293) were obtained for cat #7355 and its alleged offspring, in order to verify paternity. The same markers, including CATAMEL and CATZFXY, were used to verify chimerism in the blood leukocytes and hair follicle samples from case #7513. The analysis was performed by an external service (VHLGenetics Laboratory, Netherlands).

### DNA sequence analysis of candidate genes (*SRY, TAC3, CYP11B1* and *LHCGR*)

PCR primer pairs were designed using the Primer3 tool (https://www.bioinformatics.nl/cgi-bin/primer3plus/primer3plus.cgi) to amplify exon 3 of the *TAC3* gene that harbored the causative SNP c.220G > A^13^, exon 7 of the *CYP11B1* gene that comprised the missense G > A SNP (Arg > Gln substitution)^14^, all eleven exons of the *LHCGR* and the entire coding sequence of the *SRY* gene (Table S2). *TAC3, CYP11B1* and *SRY* fragments were amplified using standard *Taq* polymerase (EurX) and *LHCGR* with PyroMark PCR kit (Qiagen). The amplicons were purified using FastAP Thermosensitive Alkaline Phosphatase (Thermo Scientific) and Exonuclease I (Thermo Scientific), and the mixture of fragments obtained using BigDye Terminator v3.1 (Applied Biosystems) was filtered on a Sephadex G-50 (Sigma) and separated on 3130 Genetic Analyzer (Applied Biosystems). Chromatograms were aligned to the reference genomic sequences (NM_001009240.1 for *SRY*, NC_058374 for *TAC3,* ENSFCAG00000018677 for *CYP11B1* and NC_018725.3 for *LHCGR*) and analyzed using SeqMan software (DNASTAR). The odds ratio test was used to detect DNA variants associated with an increased risk of DSD.

### Droplet digital PCR (ddPCR)

Droplet digital PCR (ddPCR) was used to estimate *SRY* gene copy number and to detect XX/XY chimerism. The ddPCR primers and probes for the *SRY* gene were designed to cover part of the coding (c.87–c.200) and 5′-regulatory (− 201 to − 300 bp upstream from ATG) regions of the *SRY* gene. The *F2* gene was used as a reference gene. The X and Y copy numbers were estimated for case #7513 through copy number analysis of *AMELX* (X-linked) and *AMELY* (Y-linked) in order to detect the XX and XY lines in leukocytes and in the *in vitro* cultured fibroblasts, following the procedure described elsewhere^25^ (Table S2). Each ddPCR reaction consisted of 20 ng of DNA, 1 × Supermix for Probes (Bio-Rad), 450nM of each primer and 250nM of each probe, and 2.5U of *Hae*III (New England BioLabs) and *EcoR*I (Thermo Scientific) restriction enzymes for *SRY* and *AMELX/AMELY* assays, respectively.

The PCR reactions were partitioned into approximately 20,000 droplets using a QX200 Droplet Generator (Bio-Rad). PCR was performed on a T100 Thermal Cycler (Bio-Rad) using the following thermal cycle conditions: denaturation at 95 °C for 10 min; 40 cycles at 94 °C for 30 s and 58 °C for 60 s (ramp rate 2 °C/s); 98 °C for 10 min, and 10 °C until reading time. Droplets were detected and analyzed on a QX200 Droplet Reader (Bio-Rad). The number of copies was calculated using the Poisson distribution with QuantaSoft Software (Bio-Rad).

### Bioinformatical analysis

Transcription factor- binding sites (TFBS) disturbed by variants in the DNA sequence were designated using PROMOv3.0.2 software^26^. The potential effect of the detected variants in the 5′UTR region within target sequences for RNA binding proteins was predicted using RBPmap software^27^. Due to location of the *LHCGR* gene on the complementary DNA strand in A3 chromosome, all bioinformatical analyses were performed for the DNA strand complementary to the reference genome.

## Results

### Initial classification of DSD cases

Among the seventeen DSD cats we studied, an abnormal sex chromosome complement was found in three cases, which were considered cases of sex chromosome DSDs. In the remaining fourteen cases, a normal male sex chromosome set (38,XY) and the *SRY* gene were observed, and they were thus considered as XY DSDs (*SRY*-positive).

### Analysis of the sex chromosome set in the DSD cases

**Figure 1:**
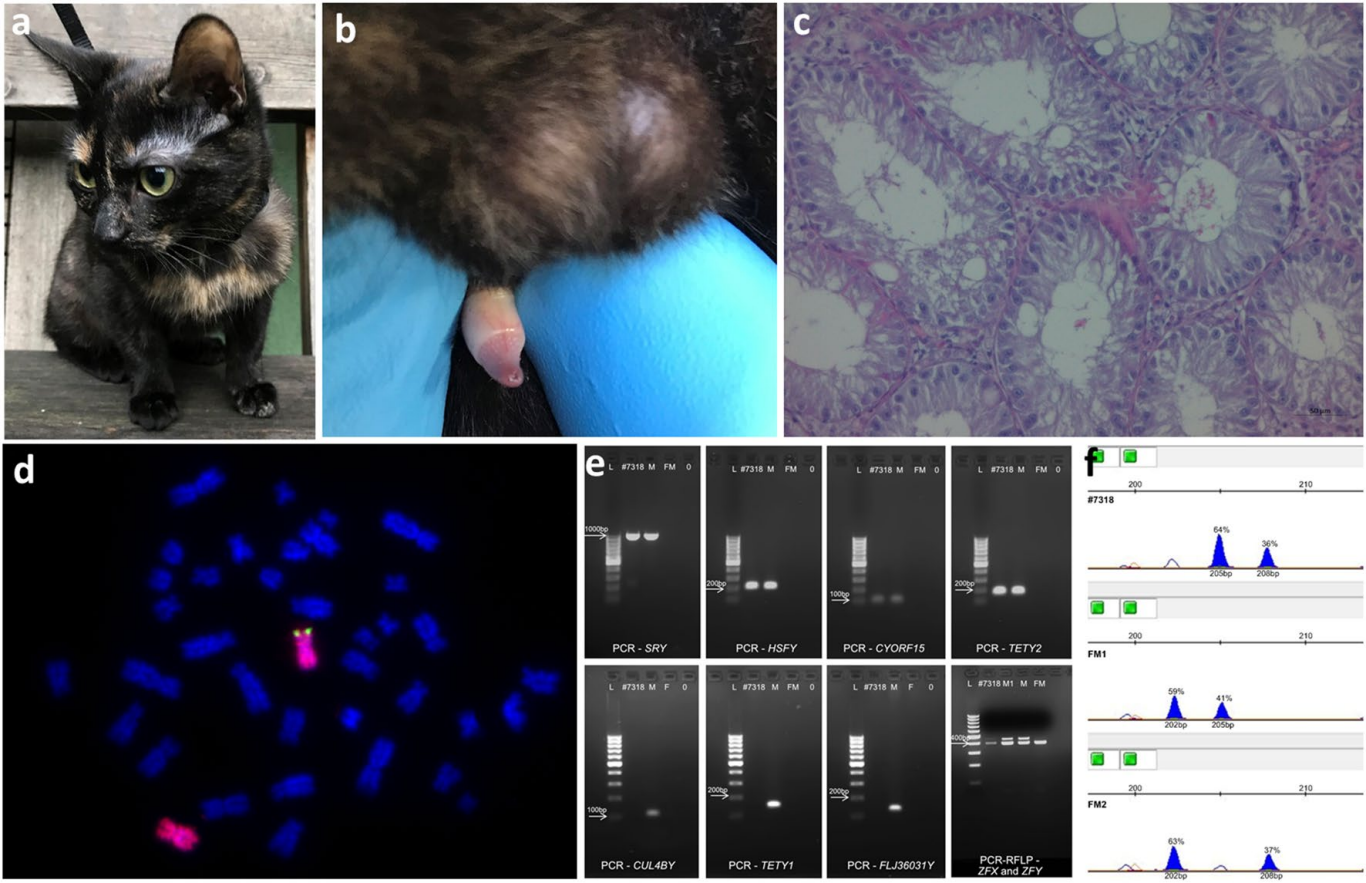
Characteristics of DSD case #7318: (**a**) tricolored coat pattern; (**b**) male external genitalia with bifid scrotum; (**c**) histology of gonads: testes with seminiferous tubules with normal Sertoli cells, normal Leydig cells, and lack of germinal cells; (**d**) FISH showing the *SRY* gene (green) on the X chromosome (red); (**e**) detection of genes translocated on the X chromosome. The Yp-linked genes were amplified for the DSD cat (*SRY, HSFY, CYORF15, TETY2, ZFY*) and the Yq-linked genes were absent (*CUL4BY*, *TETY1* and *FLJ36031Y*). L: 100–1000 bp ladder, DSD case #7318, M: control male; FM: control female; 0: negative control; (**f**) random inactivation pattern of X chromosomes in case #7318 and two control females, FM1 and FM2.

Cytogenetic analysis of three sex chromosome DSD cases revealed different abnormalities. In case #7318 (Fig. 1a–d), a translocation between X and Y chromosomes was identified. Analysis of the Giemsa-stained chromosome preparations showed normal morphology of the X chromosomes (38,XX, not shown), but the FISH technique revealed on a short arm of an X chromosome a hybridization signal produced by *SRY*-specific probe. The signal was visible in all metaphase spreads, so the karyotype was described as 38,XX^*SRY*+^. Further molecular analysis confirmed the presence of several genes (*SRY, ZFY, HSFY, CYORF15* and *TETY2*) located on a short arm of the Y chromosome (Yp), while no genes from the long Yq arm (*CUL4BY, TETY1* and *FLJ36031Y*) were detected (Fig. 1e). Additionally, the activation/inactivation status of both X chromosomes was determined from cytosine methylation status in CAG repeats in exon 1 of the androgen receptor (*AR*) gene. It was shown that both X and X^*SR*Y+^ chromosomes were randomly inactivated. The X-inactivation ratio was 64:36 in the study case and similar in two control females—59:41 and 63:37, respectively (Fig. 1f).

**Figure 2:**
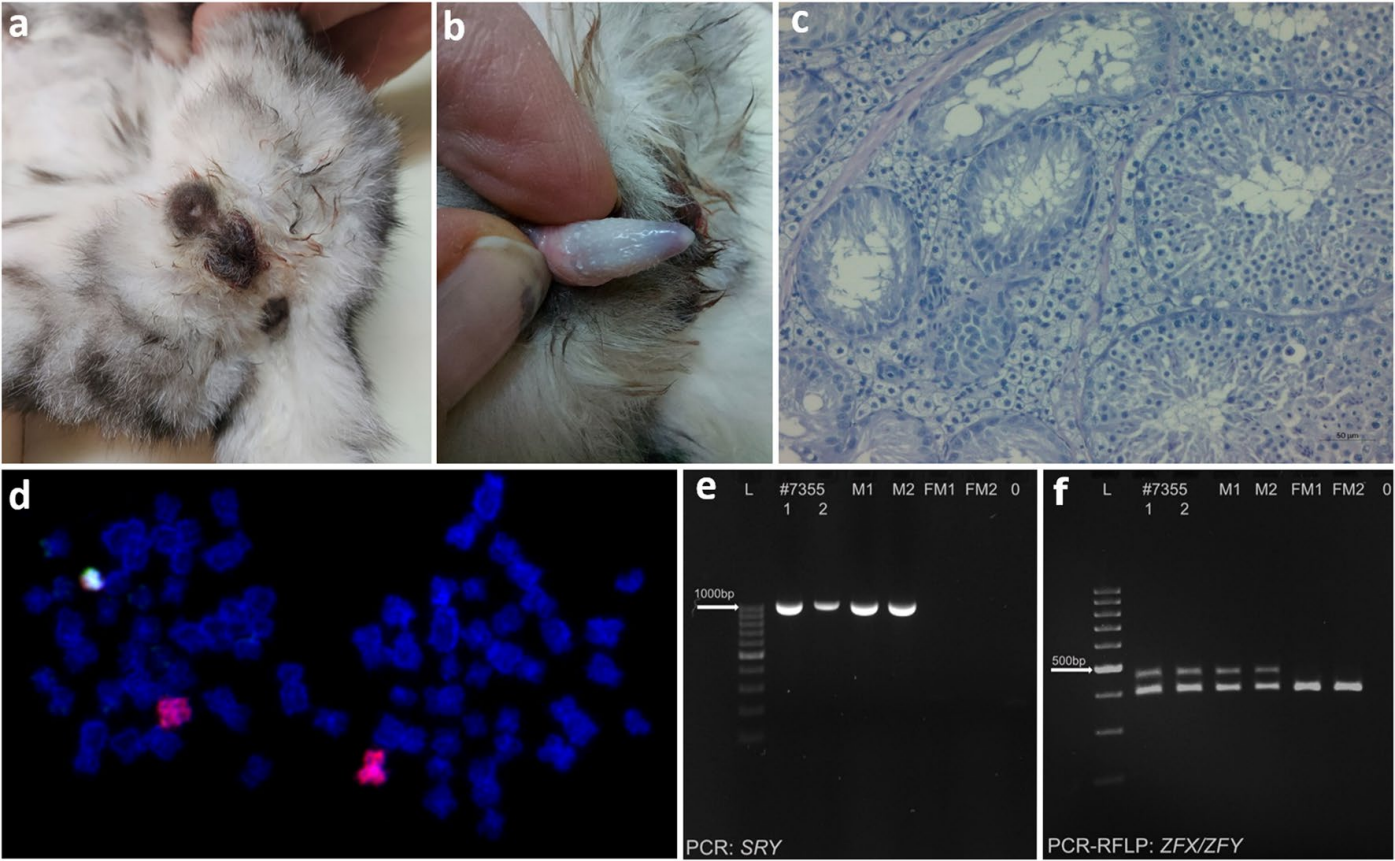
Characteristics of DSD case #7355: (**a, b**) normal male external genitalia; (**c**) histology of gonads: testes with degenerated spermatogenesis and rare sperms; (**d**) FISH with X (red) and Y (green) chromosome painting probes showing two cell lines: normal (38,XY) and monosomic (37,X); detection of Y-linked genes in blood leukocytes: (**e**) *SRY*: 1022 bp band detected by PCR, and (**f**) *ZFY*: 448 bp band (391 bp band represents the X-linked *ZFX* gene) detected by PCR–RFLP, L: 100 – 1000 bp ladder, lanes 1, 2: case #7355 (duplicated), M1, M2: control males; FM1, FM2: control females; 0: negative control.

Two cell lines were found in the second sex chromosome DSD case (#7355, Fig. 2a–c). Of the thirty Giemsa-stained metaphase spreads, 90% had a normal male chromosome complement (38,XY) and 10% were monosomic (37,X). This observation was confirmed by FISH analysis (Fig. 2d). Since these cell lines were also detected in fibroblasts, the mosaic karyotype 37,X/38,XY was designated. Molecular analysis confirmed the presence of the X (*ZFX*) and Y-linked genes (*SRY* and *ZFY*) in blood leukocytes (Fig. 2e–f). The STR analysis of this case and his offspring confirmed paternity.

**Figure 3:**
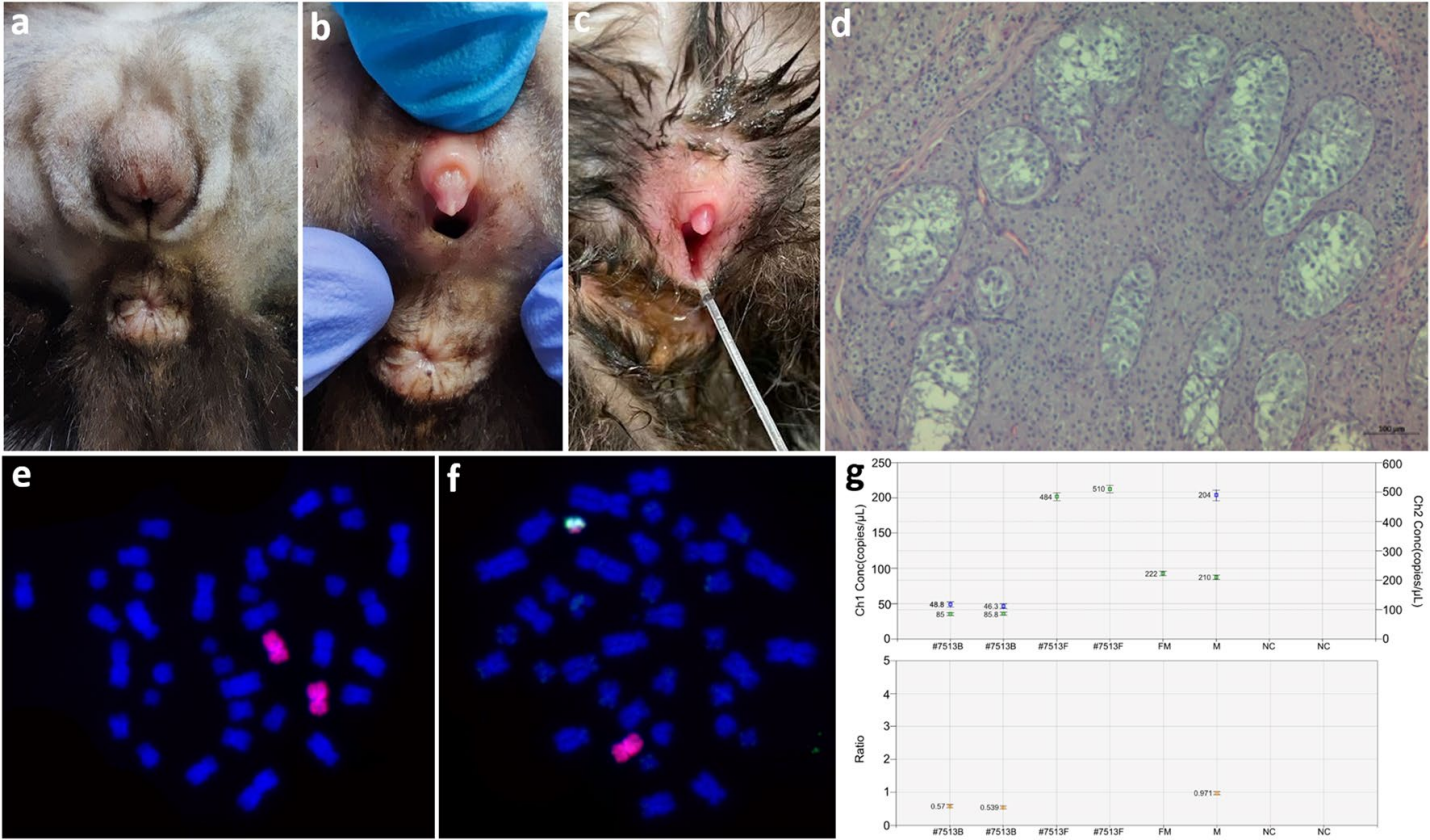
Characteristics of DSD case #7513: (**a, b, c**) ambiguous external genitalia: rudimentary penis and vulva; (**d**) histology of gonads: testes with sparse seminiferous tubules, hypertrophic Sertoli cells, numerous Leydig cells and lack of germinal epithelial cells; FISH with X (red) and Y (green) chromosome painting probes showing two cell lines: 38,XX (**e**) and 38,XY (**f**) in lymphocytes; (**g**) estimation of the Y/X ratio based on proportion of *AMELY* and *AMELX* genes in blood leukocytes (#7513B) and fibroblasts (#7513F). The upper chart shows amplification signals from chromosome X (green color) and Y (blue color). In fibroblasts of case #7513 (#7513F) the signal was detected from chromosome X only. Both signals were detected in blood leukocytes (#7513B). On the lower chart, an Y/X ratio of approximately 0.5 was detected in blood leukocytes (#7513B), indicating the presence of two cell lines. In fibroblasts of DSD case #7513 (#7513F) and in a control female (FM), the lack of Y chromosome resulted in a Y/X ratio of = 0, while a Y/X ratio of approximately 1 was detected in a control male (M); NC: negative control.

The third case (#7513), analyzed using FISH, had two cell lines, 38,XX—41%, [95% confidence interval (33%;50%)] and 38,XY—59%, [95% confidence interval (50%; 67%)], in leukocytes and a single cell line (38,XX) in fibroblasts (Fig. 3a–f). Molecular analysis confirmed the presence of Y-linked genes (*SRY*, *ZFY*) in blood, while the Y-linked genes were not present in the DNA from fibroblasts (Fig. S1). The use of ddPCR facilitated a precise estimation of the Y/X ratio and the result (ratio = 0.5) was in agreement with that of cytogenetic analysis (Fig. 3g). The STR analysis showed the presence of additional alleles for autosomal markers in DNA isolated from blood that were not present in DNA from hair follicles, confirming XX/XY leukocyte chimerism (Fig. S2). In case of two markers from sex chromosomes (CATZFXY and CATAMEL), Y-derived amplicons (for *ZFY* and *AMELY*, respectively) were present in blood while absent in DNA from hairs (Fig. S2).

### Analysis of XY DSD cases

**Figure 4:**
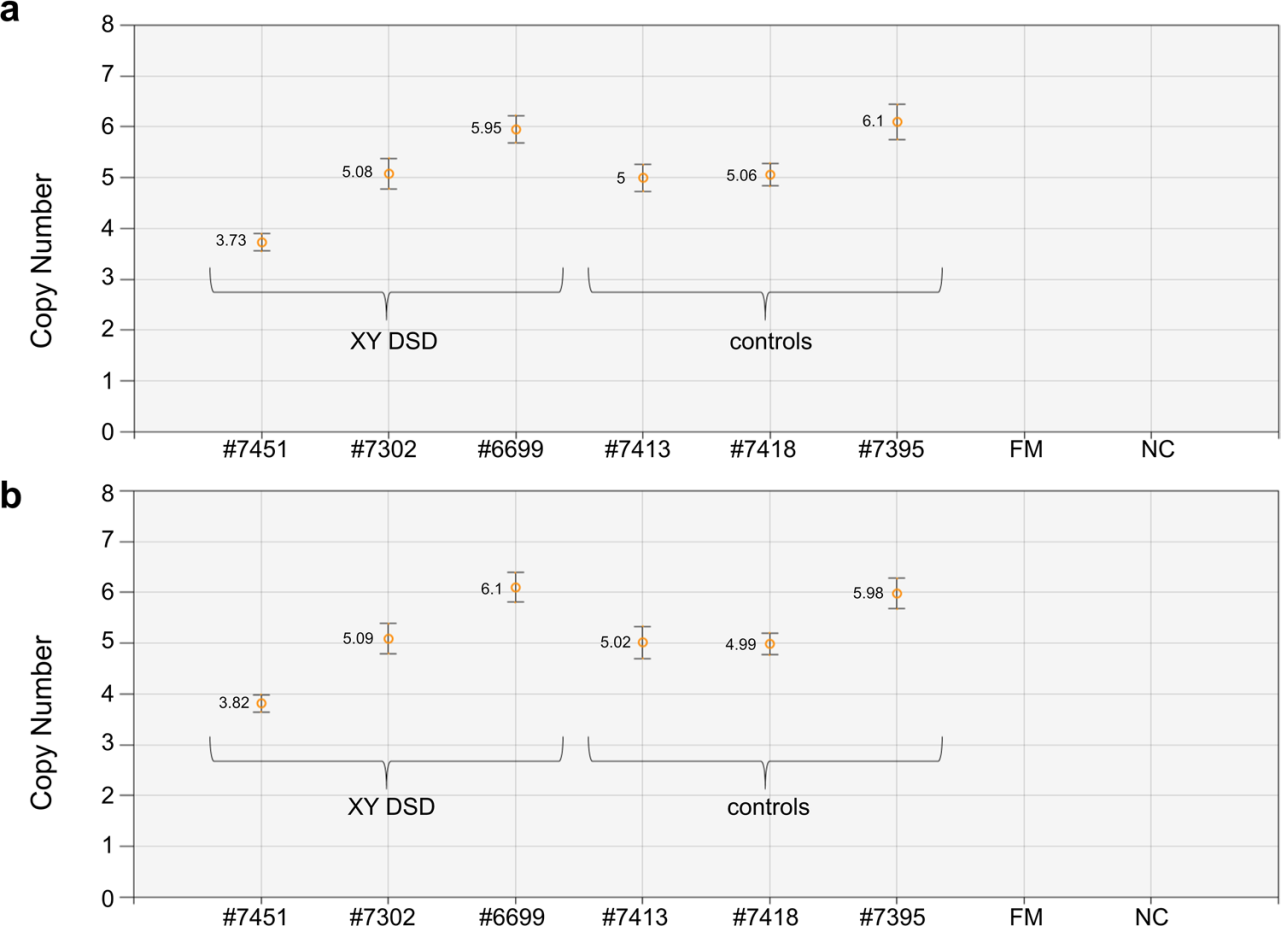
Variability of *SRY* copy number in XY DSD and control cats: (**a**) assay targeting *SRY* coding sequence; (**b**) assay targeting 5′-flanking region of *SRY*. FM: control female; NC: negative control.

The remaining fourteen XY DSD cases were investigated in order to search for DNA variants in four candidate genes (*SRY*, *TAC3, CYP11B1* and *LHCGR*). The sequencing of the entire *SRY* coding region revealed the presence of a known missense c.389G > C (p.Arg130Thr) substitution, which occurred in XY DSD and control cats with high frequencies of 0.71 and 0.58, respectively (Table 2, Table S3). We did not detect any new DNA variants in the coding sequence of the *SRY* gene, so we concluded that variants of this gene are not responsible for DSD phenotype in the study group. In the next step, we used ddPCR to examine the variability of the *SRY* gene copy number in all XY DSD cats and control males. The majority of the cats had five copies of *SRY* (Table 2), but a single case with six copies was identified in the DSD and control cohorts, and a single DSD cat (#7451) had four copies (Fig. 4a, Table S3). We performed additional analysis using an assay that targeted the 5′-flanking region of feline *SRY* and found that the copy number of the *SRY* coding sequence and the 5′-flanking region was fully consistent in all cats (Fig. 4b, Table S3). This observation suggests that all *SRY* gene copies are functional.

**Table 2:** Molecular analysis of the *SRY* gene in XY DSD and control cats. Genotype frequencies for a known c.389G > C SNP and *SRY* copy number.

Group	Frequency of genotypes for *SRY* c.389G > C SNP (p.Arg130Thr)		*SRY* copy number					
			Coding sequence			5′-flanking region		
	GG	GC	4	5	6	4	5	6
XY DSD (n = 14)	0.29 (n = 4)	0.71 (n = 10)	0.07 (n = 1)	0.86 (n = 12)	0.07 (n = 1)	0.07 (n = 1)	0.86 (n = 12)	0.07 (n = 1)
Controls (n = 38)	0.42 (n = 16)	0.58 (n = 22)	0 (n = 0)	0.97 (n = 37)	0.03 (n = 1)	0 (n = 0)	0.97 (n = 37)	0.03 (n = 1)

We next searched for the known causative nonsynonymous SNP (c.220G > A, p.Val74Met) in the *TAC3* gene^13^. This variant was not detected in the studied DSD cohort, with all the animals having a normal GG genotype. In DSD cats we did not find the previously reported causative missense variant (Arg > Gln) in exon 7 of the *CYP11B1*^14^*.* A single DSD case (#6664) was heterozygous for a synonymous c.1170G > A (p.Val390Val) substitution but this variant was assumed as not being responsible for DSD due to lack of its effect on the CYP11B1 amino acid sequence.

**Figure 5:**
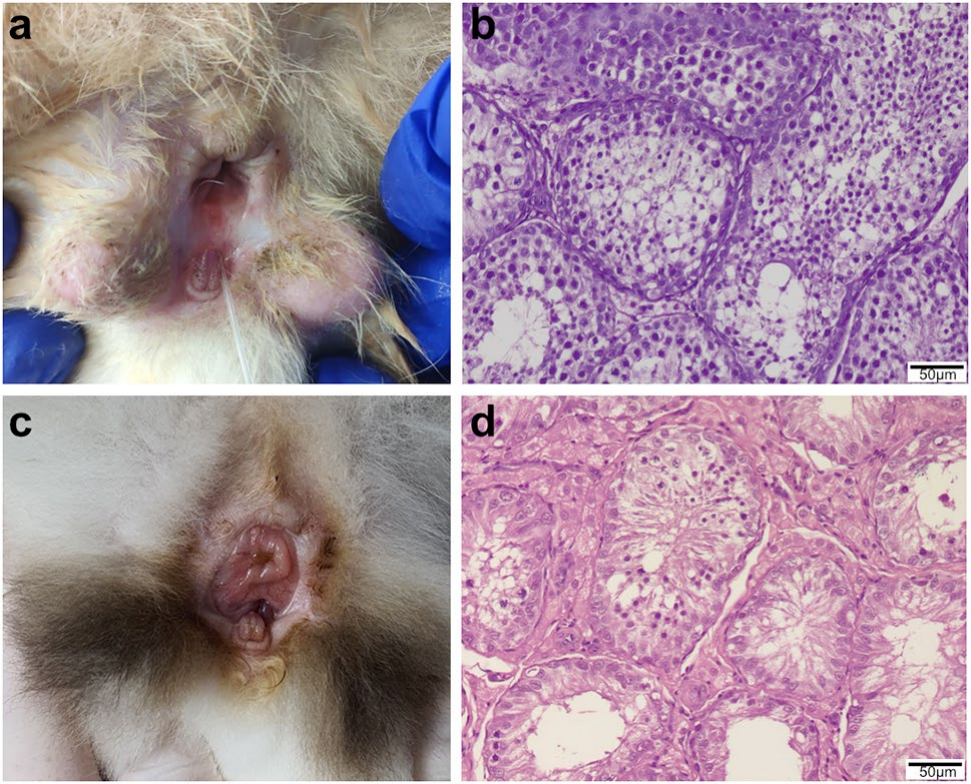
Phenotypes of two DSD cats which were homozygous for the g.70455376delC variant in 5′UTR of the *LHCGR* gene. Case #7302: (**a**) external genitalia with atypical penis, hypospadias, abnormal mucosa in the perineal area, and bifid scrotum (**b**) histological examination of gonads: numerous seminiferous tubules and Sertoli cells, sperm, and decreased number of Leydig cells; Case #7455: (**c**) external genitalia with abnormal penis, hypospadias, atypical mucosa, and bifid scrotum, (**d**) examination of gonads revealed asymmetrical testes with numerous seminiferous tubules, in larger testis with sperm and in smaller testis without sperm.

Finally, the entire coding sequence, as well as the 5′-regulatory region of the *LHCGR* gene, was analyzed. We found a total of nine novel variants in the sequence and their positions in A3 chromosome are shown in Table 3. A C > G substitution (g.70455430C > G) was found in the 5′-flanking region, 23 bp upstream of the transcription start site (TSS), and a C deletion (g.70455376delC) was found 24 bp upstream of the first ATG codon in the 5′ untranslated region (5′UTR). We identified in the coding sequence two nonsynonymous variants, g.70455334C > T in exon 1 (p.Ala7Thr) and g.70400960C > T in exon 11 (p.Ala690Thr), and two silent SNPs, g.70429346A > G in exon 5 and g.70410701A > G in exon 9. We also found three intronic SNPs that were captured because of their location in the region contiguous to the examined exons (Table 3). We assumed that the nonsynonymous variants and the polymorphisms located in 5′-regulatory region were potentially pathogenic, as they might affect the structure of the encoded protein or affect the expression of this gene. The bioinformatic analysis of putative consensus sequences for transcription factors (TFs) in the 5′-flanking region revealed that the G variant located 23bp upstream of TSS may disturb the binding of ZF87, PURA, and PURB. Further *in silico* analysis showed that the g.70455376delC variant in 5′UTR impairs the binding of following RNA-binding proteins (RBPs): RBM4, RBM4B, SRSF10, SRSF2, SRSF8. To test whether these potentially pathogenic variants might be associated with DSD, we compared their distributions in the XY DSD and control cohorts. The odds ratio (OR) test showed that the *del* variant in the 5′UTR (g.70455376delC) was significantly associated with an increased risk (*p* = 0.0467, OR = 2.9778) of DSD (Table 4). Interestingly, the only two cats with the *del/del* genotype (#7302 and #7455; Table S4) had similar phenotypes, including penile malformation, hypospadias, bifid scrotum, and mucosa abnormally located in the perineal and anal areas (Fig. 5a–d).

**Table 3:** Polymorphism detected in the feline *LHCGR* gene.

Location in *LHCGR*	Genomic position^a^	Effect on amino acid sequence
5′-flanking region/-23 upstream of TSS	A3: g.70455430C > G	None
5′UTR/exon 1/-24 upstream of ATG	A3: g.70455376delC	None
Exon 1	A3: g.70455334C > T	p.Ala7Thr
Intron 3	A3: g.70434326T > C	None
Exon 5	A3: g.70429346A > G	None (p.Asn151Asn)
Intron 5	A3: g.70429330G > A	None
Exon 9	A3: g.70410701A > G	None (p.Pro239Pro)
Intron 10	A3: g.70407203T > C	None
Exon 11	A3: g.70400960C > T	p.Ala690Thr

^a^Positions in the *LHCGR* gene according to Felis_catus_9.0 chromosome A3 (NC_018725.3) reference sequence.

**Table 4:** Frequencies of genotypes and alleles for variants in the *LHCGR* gene analyzed in XY DSD cases versus control group.

Polymorphism (location)	XY DSD (n = 14)	Controls (n = 38)	Odds ratio (OR) and *p*-value
**g.70455430C > G (5′-flanking, -23 bp upstream to TSS)**	**Genotype frequency**		OR = 2.1429 *p* = 0.2287
CC	0.71	0.82	
CG	0.21	0.18	
GG	0.07	0	
	**Allele frequency**		
C	0.82	0.91	
G	0.18	0.09	
**g.70455376delC (5′UTR, exon 1)**	**Genotype frequency**		OR = 2.9778 *p* = 0.0467
ins/ins	0.57	0.76	
ins/del	0.29	0.24	
del/del	0.14	0	
	**Allele frequency**		
ins	0.71	0.88	
del	0.29	0.12	
**g.70455334C > T p.Ala7Thr** **(exon 1)**	**Genotype frequency**		OR = 1.1325 *p* = 0.9398
CC	1	0.97	
CT	0	0.03	
TT	0	0	
	**Allele frequency**		
C	1	0.99	
T	0	0.01	
**g.70400960C > T p.Ala690Thr (exon 11)**	**Genotype frequency**		OR = 2.6825 *p* = 0.2142
CC	0.86	0.68	
CT	0.14	0.29	
TT	0	0.03	
	**Allele frequency**		
C	0.93	0.83	
T	0.07	0.17

## Discussion

Extension in our knowledge of feline genome organization has facilitated the identification of numerous gene mutations that cause hereditary diseases^28^. The OMIA database (https://www.omia.org/home/) includes over one hundred causative mutations, but only two of these are associated with DSDs. Despite this, abnormalities of sex chromosome complement in DSD male cats are quite frequently reported (for a review, see Szczerbal and Switonski^1^). This could be due to the genetic background of the orange coat color, which is controlled by a dominant X-linked gene. In females heterozygous at this locus, a tricolor coat is observed due to the random inactivation of one X chromosome. The occurrence of the tricolor coat pattern in males suggests an abnormality in the sex-chromosome complement. In male cats, tortoiseshell and calico coats are frequently seen to cooccur with XXY syndrome^7,8^ but the rare phenomenon of whole-body chimerism may also lead to a tricolor coat, as shown in a fertile tortoiseshell male cat with true chimerism (38,XY/38,XY)^29^. It should be mentioned that this type of coat can also be observed in DSD virilized females with leukocyte XX/XY chimerism and in carriers of X/Y translocation leading to the transfer of *SRY* gene from the Y to the X chromosome (38,XX^*SRY*+^) or even in very rare cases of 38,XX^*SRY*−^ males^5,12^.

We have here described the second case of X/Y translocation; to best of our knowledge, this type of abnormality in domestic animals has only been found to date in two cats—the present case and a cat reported earlier by Szczerbal et al.^5^ It is worth mentioning, that a similar abnormality (46,XX^*SRY*+^) exists in humans and is also rare (1:20,000 newborn boys); this is called de la Chapelle syndrome^30^. However, in humans it is caused by unequal crossing over between the X and Y chromosomes, since the *SRY* is located very close (~ 11 kb) to the pseudoautosomal region (PAR) in the Y chromosome. Feline *SRY* is, however, located approx. 2 Mb from the PAR as it was presented by Szczerbal and Switonski^1^.

The second chromosome abnormality described here was a mosaicism (37,X/38,XY) with a predominant 38,XY cell line (90%). Interestingly, the same type of mosaicism has previously been described in a Bengal cat with cryptorchidism and Müllerian duct remnants^9^, but the proportion of cell lines was different at 37,X[96%]/38,XY[4%]. This could be responsible for the differing phenotype of our case, which displayed feminine behavior, testes with degenerated spermatogenesis, and rare sperm. Interestingly, this cat has a single offspring, as we confirmed with molecular parentage testing. In the cat described by Balogh et al.^9^, the testes were not capable of spermatogenesis. Since both cases were recognized as males, it can be supposed that the observed X/XY karyotype was the result of Y chromosome instability during mitotic cell divisions. Such a mechanism was probably also responsible for the disorder of sexual development in a cat with chromosome mosaicism 37,X/38,X,der(Y)^6^.

The sexual phenotype of the third case, which carried leukocyte XX/XY chimerism, was similar to the previous one reported by Szczerbal et al.^7^ Both cats had a vulva, a rudimentary penis, and abdominally located testicles. The proportion of XX/XY cell lines was also similar (41/59 and 35/65, respectively), but it is known from studies of such abnormalities in other species (such as cattle and dogs) that there is no correlation between the percentage of XY cells and the extent of the virilization^31,32^. We also used ddPCR to quickly and reliably detect the XX and XY cell lines in cats; this approach has a proven usefulness for diagnosis of these type of sex chromosome DSD in cattle and pigs^25,33^.

Of the DSD cats we examined, the majority were XY DSD, and most presented a variable degree of gonadal dysgenesis (GD). This can be caused by or associated with numerous gene variants, as has been observed in humans^34^. Of these, mutations of *SRY* are responsible for at least 15% of cases of GD. In the present study, we found a common missense variant of this gene, which was described earlier by Nowacka-Woszuk et al.^10^ We also examined the variability of the *SRY* copy number. It has previously been reported that the feline genome contains a single copy of *SRY*^23^, but we have shown here for the first time that the copy number in fact varies between four and six, with a modal number of five. This variability did not show any association with the DSD phenotype in cats. It has recently been reported that a smaller number of *SRY* copies (two rather than three) may be associated with DSD in Yorkshire terrier dogs^18^.

The next selected candidate gene was *TAC3*, due to the deleterious effect of missense substitution (c.220G > A, p.Val74Met) on testicle development in cats^13^. We sequenced exon 3 harboring this variant in a cohort of fourteen XY DSD cats, but all of them had normal genotype (GG). Mutations of *TAC3* are also quite rare causes of human XY DSD, with the OMIM database containing only a few reports of hypogonadotropic hypogonadism 10, with or without anosmia, caused by mutation of this gene (OMIM #614839). Small testicles and micropenis are observed in such patients^35,36^. On the other hand, *CYP11B1* mutations may lead to impaired biosynthesis of cortisol and altered level of androgens. In humans they are implicated in 5-8 % of cases of congenital adrenal hyperplasia (CAH), but in males their clinical manifestations usually do not involve external genitalia^37^. Interestingly, a single cat was described previously with symptoms of CAH, including abnormalities of the genitourinary system, caused by 11β-hydroxylase deficiency due to a missense substitution in exon 7 of *CYP11B1*^14^. We showed that this variant was not present in the analyzed DSD cats.

The most interesting molecular observations concerned the *LHCGR* gene: of nine novel DNA variants, the deletion of G nucleotide, present in 5′UTR, showed an association with XY DSD phenotype. It is worth mentioning that the two DSD cats with the *del/del* genotype had similarly abnormal sexual phenotypes. Bioinformatic analysis of the sequence harboring this variant revealed that the *del*G variant impairs binding of the regulatory RNA-binding proteins, which may affect the expression of *LHCGR*. Studies of knock-out mice with disrupted *LHCGR* promoter regions showed underdevelopment of internal and external genitalia, including micropenis, abdominal testes, hypertrophy of Leydig cells, and spermatogenic arrest at the round spermatid stage^38^. The importance of *LHCGR* mutations has been documented by studies of numerous causative variants, mainly missense substitutions, in humans^19^.

Our study showed that XY DSD cats form a heterogeneous group and that there are no common pathogenic mutations. Studies on large cohorts of affected animals with the same abnormal phenotype should thus be conducted. It can be anticipated that a comprehensive method for analyzing entire genomes—such as dense genotyping array, whole exome sequencing (WES)^39,40^, or whole genome sequencing (WGS)—would greatly facilitate the search for causal variants for XY DSD in cats.

## Conclusions

In this study, we analyzed a large cohort of cats with complex abnormalities of sex development. Among the studied cases, sex chromosome DSDs were quite frequent, with three different chromosomal abnormalities found (38,XX^*SRY*+^, 37,X/38XY, XX/XY leukocyte chimerism). The second group of affected animals consisted of XY DSD cohort, and we were able to exclude known mutations in *TAC3* and *CYP11B1* as a cause of their DSD phenotype. On the other hand, we showed that a single nucleotide deletion in the regulatory sequence of the *LHCGR* gene is a promising marker associated with DSD phenotype in some cases. Finally, we detected for the first time an elevated number of *SRY* copies in normal and DSD cats, but we did not observe an association between the copy number and DSD phenotype. Our study thus extends the knowledge of the genetic background of DSD in cats.

## Supplementary Information

Supplementary Information.

## Data Availability

The datasets generated and analyzed during the current study are available in the European Variation Archive (EVA) repository under accession no. PRJEB55345 (https://www.ebi.ac.uk/eva/?eva-study=PRJEB55345).
