# Supplementary material for: Cytogenetic and molecular insight into the genetic background of disorders of sex development in seventeen cats
Source: Sci Rep. 2022 Oct 24;12:17807. doi: 10.1038/s41598-022-21718-y (PMC9592617; doi:10.1038/s41598-022-21718-y)
Supplement: Supplementary file 1 — Supplementary Information. [file 41598_2022_21718_MOESM1_ESM.pdf]

Supplementary materials

Cytogenetic and molecular insight into the genetic background of disorders of sex development in seventeen cats, by Stachowiak et al.

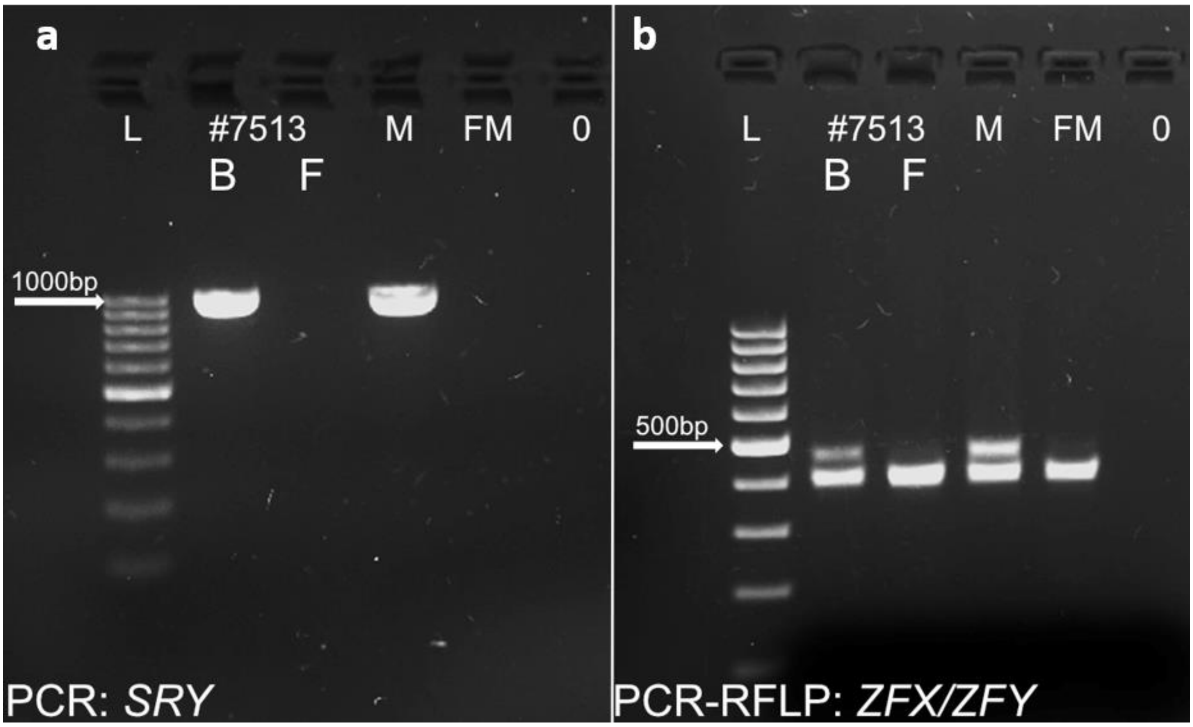

Figure S1. Detection of Y-linked genes in blood (B) and fibroblasts (F) of DSD case #7513. (**a**) *SRY* – 1022 bp band, and (**b**) *ZFY* – 448 bp band (391 bp band represents the X-linked *ZFX* gene). L – 100 – 1000 bp ladder, M – control male, FM – control female, 0 – negative control.

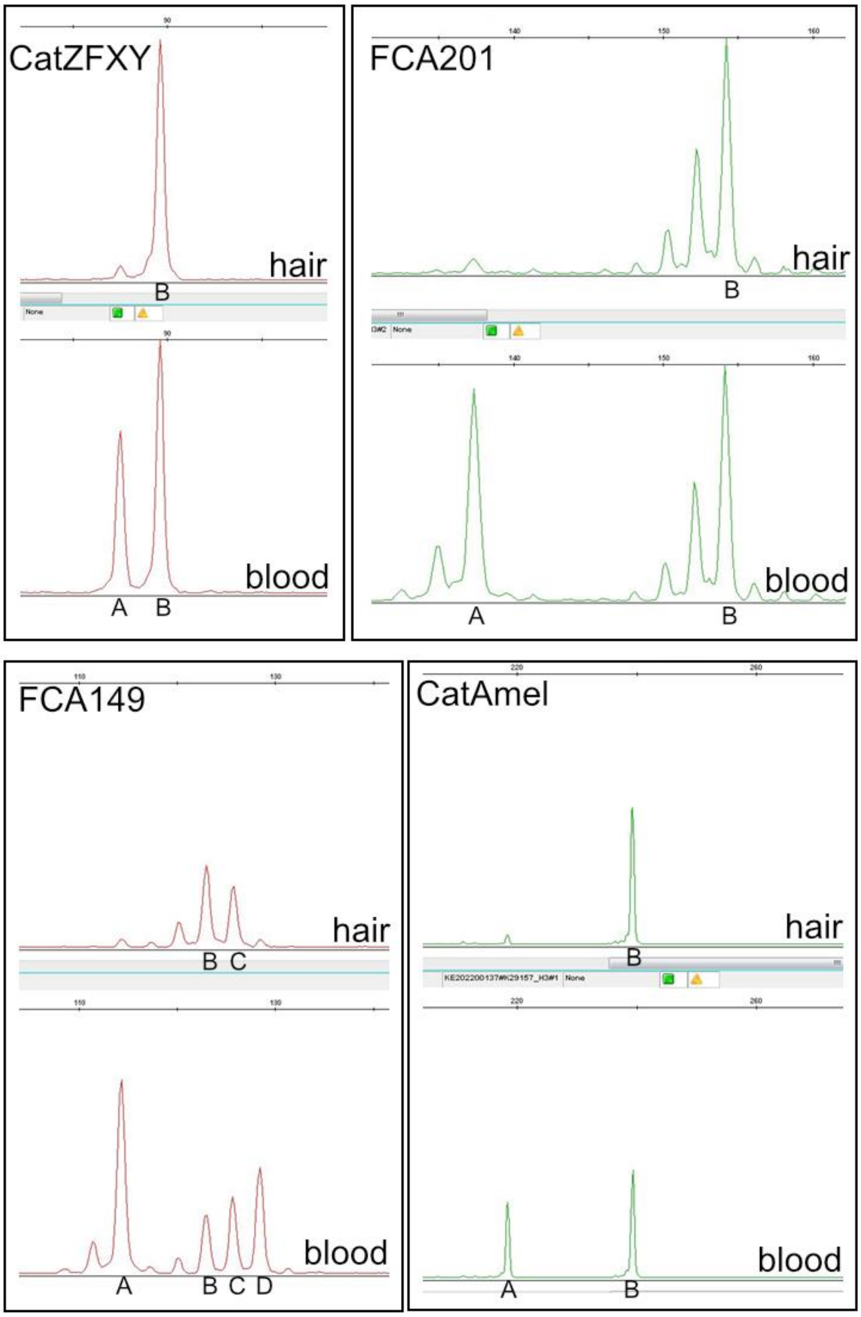

Figure S2. Analysis of STR markers, CatZFXY, FCA201, FCA149, and CatAmel in DNA isolated from hair follicles and blood of DSD case #7513 showed additional alleles in DNA from blood that were not present in DNA from hair follicles, confirming the leukocyte chimerism. A,B,C,D – alleles.

Table S1. Control males analyzed in the study.

	Cat (lab ID)	Breed	*SRY*
1	#7361	Domestic shorthair	+
2	#7362	British	+
3	#7363	Maine coon	+
4	#7364	Domestic shorthair	+
5	#7365	Ragdoll	+
6	#7367	Domestic shorthair	+
7	#7371	British longhair	+
8	#7372	Maine coon	+
9	#7378	Domestic shorthair	+
10	#7383	Turkish angora	+
11	#7384	Turkish angora	+
12	#7387	Neva masquerade	+
13	#7388	Domestic shorthair	+
14	#7390	Domestic shorthair	+
15	#7392	British	+
16	#7393	British	+
17	#7394	Maine coon	+
18	#7395	Domestic shorthair	+
19	#7396	Domestic shorthair	+
20	#7399	Domestic shorthair	+
21	#7400	Domestic shorthair	+
22	#7405	Bengal	+
23	#7408	Maine coon	+
24	#7409	Maine coon	+
25	#7412	Domestic shorthair	+
26	#7417	Norvegian forest cat	+
27	#7418	British shorthair	+
28	#7421	Domestic shorthair	+
29	#7422	Domestic shorthair	+
30	#7424	Ragdoll	+
31	#7429	Domestic shorthair	+
32	#7432	Domestic shorthair	+
33	#7435	Domestic shorthair	+
34	#7376	Domestic shorthair	+
35	#7419	Domestic shorthair	+
36	#7427	British	+
37	#7434	Domestic shorthair	+
38	#7413	Domestic shorthair	+

Table S2. PCR primers and assays used in the study.

Gene	Primer sequence 5' → 3'	Amplicon (bp)	Annealing temperature (°C)
**X inactivation status**			
AR (exon 1)	F: atccacgttgtccctgct	209	60
	R: 6-FAM-cagtggcctcccttgttct		
**Detection of Y-linked genes or sequencing of candidate genes**			
SRY (coding sequence)[4]	F: aactttgctacccaccaacc	1022	58
	R: caatctgcggaaaactggat		
ZFX/ZFY[20]	F: ataatcacatggagagccaccagct	448	58
	R: gcacttctttggtatctgagaaagt		
TETY1[21]	5' cagatgtgcaggggagcac<br>5' aatatatatgtcctccaataaacgatg	150	58
TETY2[21]	5' aaatgacccagcttccattg<br>5' gctttgggcatcagtctagc	178	58
CUL4BY[21]	5' ataaacccctgccactgga<br>5' tcttccaagctgtatttaacagaga	106	58
CYORF15[21]	5' tgtggttaggtgaaaacacacc<br>5' tgattcctatgaccaggcttc	100	58
HSFY[21]	5' tggatcagcatgccatttta<br>5' ctggctgacacagcaggata	211	58
FLJ36031[21]	5' gcctgaggagaaggtgttgt<br>5' gcacctttctctggctgacc	128	58
TAC3 (exon 3)	F: tttctaacaagcccacagca	424	62
	R: attcaccaagccatctccag		
CYP11B1[14]	F: aggctcagggaactttgtctg	413	60
	R: gtttcgacccagggagtagag		
LHCGR (exon 1)	F: ggttgaggcagagcacagt	500	59
	R: gggatggcagagacagtcc		
LHCGR (exon 2)	F: aactccggtgtgtctgatcc	441	62

	R: ctggggtttcttgaacacaa		
LHCGR (exon 3)	F: gagatggcagcacttgatga	491	61
	R: gctacttggcaggactccac		
LHCGR (exon 4)	F: gcttcatagagtggggtgga	510	62
	R: cctggtggctgacaagaaat		
LHCGR (exons 5 and 6)	F: gtcccagcccctagatcttt	679	62
	R: tgggtcagttggttgttgtg		
LHCGR (exon 7)	F: tgaatgttgcatacgccagt	454	62
	R: agagggaggctgtgagatga		
LHCGR (exon 8)	F: caatccccttttccctcta	333	62
	R: cagtgcccccagttagtgtt		
LHCGR (exon 9)	F: attagggggaaggcaagatg	448	62
	R: tttcgggaagaaacaggatg		
LHCGR (exon 10)	F: ggagcccacacaaaacactt	441	62
	R: tttgcaaacatccctccaat		
LHCGR (exon 11)	F: actgaggctgttgtggcttt	873	62
	R: gccatcagctctggattttg		
LHCGR (exon 11)	F: acgctattcagctggaccaa	859	62
	R: gcaatggtcatggacaacac		
***SRY* and *AMELX/AMELY* copy number (ddPCR)**			
SRY (coding sequence)	F: ttgcacggagagtcctacct	114	58
	R: tctcgagaccacaccatgaa		
	Probe: FAM-tgtgaaaccagaggaaaggg-BHQ1		
SRY (5’-flanking region)	F: gagacttaagcgtggcggat	100	58
	R: ccgaagcccaacctaccatt		
	Probe: FAM-gcagtgagccgagcatgaacct-BQH1		
F2 (reference gene)	F: agcgagaaccaggacttcaa	98	58
	R: ccggacacataacaccacac		
	Probe: HEX-ctggagaagaacttctgccg-BHQ1		
AMELX	F: tcagtcccgctttttcagtt	107	58
	R: agtcagagaggccgagcag		

	Probe: HEX-tcctggtttaagccctgatg-BHQ1		
AMELY	F: caagatgtttctcagtcctgct	90	58
	R: gaggccaaataggagtgtgc		
	Probe: FAM-gtttaagcctgcattgtccc-BHQ1		

References for Table S2:

[4] Szczerbal, I. *et al.* X monosomy in a virilized female cat. *Reprod. Domest. Anim.* **50**, 344–348 (2015).

[14] Owens, S. L. *et al.* Congenital adrenal hyperplasia associated with mutation in an 11β-hydroxylase-like gene in a cat. *J. Vet. Intern. Med.* **26**, 1221–1226 (2012).

[20] Senese, C., Penedo, M. C., Shiue, Y. L., Bowling, A. T. & Millon, L. V. A HaeIII PCR-RFLP in the ZFY/ZFX genes of horses. *Anim. Genet.* **30**, 390–391 (1999).

[21] Murphy, W. J. *et al.* Novel gene acquisition on carnivore Y chromosomes. *PLoS Genet.* **2**, e43 (2006).

Table S3. Results of the molecular analysis of *SRY* gene in XY DSD and control cats.

	Cat (lab ID)	Breed	Genotype for *SRY* c.389G>C SNP (p.Arg130Thr)	*SRY* coding sequence copy number	*SRY* 5'-flanking region copy number
**XY DSD cats**					
1	#6533	Persian	gc	5	5
2	#6664	Domestic shorthair	gc	5	5
3	#6680	Domestic shorthair	gc	5	5
4	#6699	Domestic shorthair	gc	6	6
5	#6721	Crossbred (Persian x Domestic shorthair)	gc	5	5
6	#6856	Domestic shorthair	gg	5	5
7	#6973	Domestic shorthair	gg	5	5
8	#7036	British shorthair	gc	5	5
9	#7145	British shorthair	gc	5	5
10	#7254	Domestic shorthair	gc	5	5
11	#7302	Domestic shorthair	gg	5	5
12	#7320	British longhair	gc	5	5
13	#7451	Domestic shorthair	gg	4	4
14	#7455	Ragdoll	gc	5	5
**Control cats**					
1	#7361	Domestic shorthair	gg	5	5
2	#7362	British	gc	5	5
3	#7363	Maine coon	gc	5	5
4	#7364	Domestic shorthair	gg	5	5
5	#7365	Ragdoll	gc	5	5
6	#7367	Domestic shorthair	gg	5	5
7	#7371	British longhair	gc	5	5
8	#7372	Maine coon	gc	5	5
9	#7378	Domestic shorthair	gg	5	5

10	#7383	Turkish angora	gc	5	5
11	#7384	Turkish angora	gc	5	5
12	#7387	Neva masquerade	gc	5	5
13	#7388	Domestic shorthair	gg	5	5
14	#7390	Domestic shorthair	gg	5	5
15	#7392	British	gc	5	5
16	#7393	British	gc	5	5
17	#7394	Maine coon	gc	5	5
18	#7395	Domestic shorthair	gc	6	6
19	#7396	Domestic shorthair	gg	5	5
20	#7399	Domestic shorthair	gc	5	5
21	#7400	Domestic shorthair	gg	5	5
22	#7405	Bengal	gc	5	5
23	#7408	Maine coon	gc	5	5
24	#7409	Maine coon	gc	5	5
25	#7412	Domestic shorthair	gc	5	5
26	#7417	Norvegian forest cat	gg	5	5
27	#7418	British shorthair	gc	5	5
28	#7421	Domestic shorthair	gc	5	5
29	#7422	Domestic shorthair	gc	5	5
30	#7424	Ragdoll	gc	5	5
31	#7429	Domestic shorthair	gg	5	5
32	#7432	Domestic shorthair	gg	5	5
33	#7435	Domestic shorthair	gg	5	5
34	#7376	Domestic shorthair	gg	5	5
35	#7419	Domestic shorthair	gg	5	5
36	#7427	British	gc	5	5
37	#7434	Domestic shorthair	gg	5	5
38	#7413	Domestic shorthair	gg	5	5

Table S4. Genotypes for polymorphisms in *LHCGR* gene analyzed in XY DSD versus control cats.

	Cat (lab ID)	g.70455430C>G (5'-flanking, -23bp upstream to TSS)	g.70455376delC (5'UTR, exon 1)	g.70455334C>T p.Ala7Thr (exon 1)	g.70400960C>T p.Ala690Thr (exon 11)
**XY DSD cats**					
1	#6533	CC	ins/ins	CC	CC
2	#6664	CC	ins/ins	CC	CT
3	#6680	CG	ins/del	CC	CT
4	#6699	CG	ins/del	CC	CC
5	#6721	CC	ins/del	CC	CC
6	#6856	CC	ins/ins	CC	CC
7	#6973	CC	ins/ins	CC	CC
8	#7036	CC	ins/ins	CC	CC
9	#7145	CC	ins/ins	CC	CC
10	#7254	CC	ins/ins	CC	CC
11	#7302	GG	del/del	CC	CC
12	#7320	CC	ins/ins	CC	CC
13	#7451	CG	ins/del	CC	CC
14	#7455	CC	del/del	CC	CC
**Control cats**					
1	#7361	CC	ins/ins	CC	CC
2	#7362	CC	ins/ins	CC	CC
3	#7363	CC	ins/ins	CC	CC
4	#7364	CC	ins/ins	CC	CT
5	#7365	CC	ins/ins	CC	CC
6	#7367	CG	ins/del	CC	CC
7	#7371	CC	ins/ins	CC	CC
8	#7372	CG	ins/del	CC	CC
9	#7378	CC	ins/ins	CC	CC
10	#7383	CC	ins/ins	CC	CC
11	#7384	CC	ins/ins	CC	CT
12	#7387	CC	ins/ins	CC	CC
13	#7388	CC	ins/del	CC	CT
14	#7390	CG	ins/del	CC	CC
15	#7392	CC	ins/ins	CC	CC
16	#7393	CC	ins/ins	CC	CC
17	#7394	CC	ins/ins	CC	CC
18	#7395	CC	ins/ins	CC	CT
19	#7396	CC	ins/ins	CC	CC
20	#7399	CG	ins/del	CC	CC
21	#7400	CC	ins/ins	CC	TT
22	#7405	CC	ins/ins	CC	CT
23	#7408	CC	ins/ins	CC	CT
24	#7409	CC	ins/ins	CC	CT

25	#7412	CC	ins/ins	CC	CT
26	#7417	CC	ins/ins	CC	CC
27	#7418	CC	ins/ins	CC	CT
28	#7421	CC	ins/ins	CC	CC
29	#7422	CG	ins/del	CC	CC
30	#7424	CC	ins/del	CC	CC
31	#7429	CC	ins/ins	CC	CC
32	#7432	CC	ins/ins	CC	CC
33	#7435	CG	ins/del	CC	CC
34	#7376	CC	ins/ins	CC	CC
35	#7419	CC	ins/ins	CT	CT
36	#7427	CC	ins/ins	CC	CC
37	#7434	CG	ins/del	CC	CC
38	#7413	CC	ins/ins	CC	CT
